# Patterns of flow drainage from varicose veins originating in the incompetent great saphenous vein

**DOI:** 10.1590/1677-5449.202200192

**Published:** 2023-01-06

**Authors:** Felipe Coelho, Maria Isabel Sarti Benatti, Mariana Cavalaro Ricciardi, Nicole Dorneli de Carvalho, Sergio Quilici Belczak, Walter Júnior Boim de Araújo, Rodrigo Gomes de Oliveira

**Affiliations:** 1 Universidade de Brasília - UnB, Brasília, DF, Brasil.; 2 Pontifícia Universidade Católica do Paraná - PUCPR, Londrina, PR, Brasil.; 3 Universidade de São Paulo - USP, São Paulo, SP, Brasil.; 4 Universidade Federal do Paraná - UFPR, Curitiba, PR, Brasil.

**Keywords:** varicose veins, saphenous vein, venous insufficiency, ultrasonography, varizes, veia safena, insuficiência venosa, ultrassonografia

## Abstract

**Background:**

Chronic venous insufficiency affects the lives of many people and therefore constitutes a public health problem. Knowledge of the drainage patterns of reflux from varicose veins secondary to incompetent saphenous veins is essential to define the best therapeutic management.

**Objectives:**

To determine the reflux drainage patterns from varicose veins originating in incompetent GSV, the prevalence of perforating veins (PV), and their relationships with symptoms.

**Methods:**

55 ultrasound reports were analyzed to determine the drainage patterns of reflux from the GSV, location and diameter of PV drainage, and staging of symptoms.

**Results:**

In 64% of the sample, reflux from varicose veins drained to PVs, in 4% reflux drained to the GSV itself, in another 4% drainage was to the small saphenous vein, and in 29% drainage was to varicose trunk veins in which no direct communication with the deep system could be identified. No associations were observed between symptoms and reflux drainage patterns or PV diameters.

**Conclusions:**

For this sample, PVs were responsible for draining flow from varicose veins in 64% of cases. Neither PV diameters nor GSV reflux patterns were associated with severity of symptoms.

## Resumo

**Introdução:**

A insuficiência venosa crônica impacta a vida de muitas pessoas, constituindo-se, assim, como um problema de saúde pública. Conhecer o padrão de drenagem do refluxo das varizes associadas à veia safena incompetente é fundamental para definir a melhor programação terapêutica.

**Objetivos:**

Determinar os padrões de drenagem do refluxo de varizes originadas da veia safena magna incompetente, a prevalência de veias perfurantes e a relação com os sintomas.

**Métodos:**

Foram analisados 55 registros ultrassonográficos de pacientes com refluxo da veia safena magna para determinar padrões de drenagem do refluxo dessa veia, pontos de refluxo das varizes, localização e diâmetro das perfurantes de drenagem e graduação dos sintomas.

**Resultados:**

O principal padrão de refluxo encontrado foi originado da junção safenofemoral com comprometimento proximal da veia safena magna. Em 64% dos pacientes, o refluxo das varizes drenou para veias perfurantes - 4% drenavam para a própria veia safena magna; em outros 4%, a drenagem era para a veia safena parva; e, em 29%, a drenavam destinava-se para varizes tronculares em que não se identificou comunicação direta com o sistema venoso profundo. Não foi observada associação dos sintomas com os padrões de drenagem do refluxo ou diâmetro das perfurantes.

**Conclusão:**

Para essa amostra, as veias perfurantes foram responsáveis pelo escoamento do fluxo oriundo das varizes em 64% dos casos. O diâmetro das veias perfurantes e o padrão de refluxo da veia safena não estiveram associados à gravidade dos sintomas.

## INTRODUCTION

Chronic venous insufficiency is a dysfunction of the venous system caused by venous valve incompetence, associated or not with obstruction of venous flow. It is a severe public health problem, not only because of its high prevalence, but also because of its socioeconomic impact, since it affects around 20% of the adult population in Western countries.^1^,^2^

The venous system of the lower limbs is divided into two compartments, either or both of which may be involved in this pathology. The deep vein system is responsible for 85% of venous drainage and the superficial vein system is responsible for the remaining 15%.

There are an average of 64 perforating veins from the ankle to the groin that directly or indirectly communicate between the two systems, enabling drainage of flow from superficial veins to deep veins and onwards in the direction of the heart.^3^

While perforating veins are numerous and variable, in general they can be classified into four groups on the basis of clinical significance - those of the foot, the medial calf, the lateral calf, and the thigh. Around 40% are associated with venous incompetence, especially those that connect to saphenous veins and tributaries.

When competent, they drain flow to the deep system without impacting on the caliber of the saphenous or tributary veins. Incompetent perforating veins transfer flow to saphenous or tributary veins, causing them to dilate.^4^,^5^

The objective of this study is to analyze patterns of drainage to the deep vein system of the flow from varicose veins originating in an incompetent great saphenous vein, the prevalence of perforating veins, and the relationships between these factors and the clinical symptoms of the patients in the sample.

## MATERIALS AND METHODS

A cross-sectional prevalence analysis study was conducted at a hospital**. U**ltrasound examination reports were analyzed from patients with great saphenous vein reflux, of both sexes, aged from 18 to 80 years. Participants were selected by convenience, recruiting patients referred for ultrasound mapping who were promptly available to be enrolled on the study from November to March of 2020, during assessments performed by a vascular surgeon qualified to conduct vascular Doppler ultrasound studies.

Patients were excluded if they did not have reflux in the great saphenous vein or if they had thrombophlebitis of the great saphenous vein, deep venous thrombosis, or signs of a previous thrombotic event involving the deep vein system.

All ultrasound examinations were performed by a certified Vascular Surgeon, using a Philips HD7 scanner (Koninklijke Philips Electronics), and following the protocol previously described by Engelhon et al.^4^

Saphenous vein reflux patterns were classified as follows:

1. Reflux at the saphenofemoral junction (SFJ) and the proximal great saphenous vein (GSV);
2. Reflux at the SFJ and reflux in multiple segments of the GSV;
3. GSV incompetence in a single segment, without SFJ compromise, combined with reflux originating in tributary or perforating veins;
4. Distal GSV incompetence;
5. Reflux at the SFJ and a GSV incompetent along its entire length;
6. Reflux originating at the SFJ and draining through tributary veins;
7. Reflux at the SFJ and the anterior accessory saphenous vein.

Patients were evaluated in the standing position, with external rotation of the limb and body weight supported by the contralateral limb. All examinations were performed using a 7-13MHz linear probe. Reflux has been defined as abnormal when retrograde flow lasts 0.500 milliseconds in the saphenous vein and abnormal perforating veins have been defined as those with an outward flow duration of 0.500 milliseconds.^4^ The perforating vein diameters were measured at the fascia transition level. The reflux drainage patterns observed were described as: to a perforator vein; to the great saphenous vein; to the small saphenous vein; or to trunk varicose veins from which it was not possible to identify drainage to the deep vein system.

The ultrasound examination reports were then reviewed to analyze the characteristics of drainage to the deep vein system of reflux from varicose tributary veins of the saphenous vein and the diameters of the perforating veins, when present.

Data on the symptoms patients reported were collected from their charts. The data collected were tabulated for statistical analysis.

### Statistical analysis

The statistical analysis was based on parametric or nonparametric tests, as appropriate to distribution of the sample. Continuous variables (age) were expressed as mean (M) and standard deviation (SD) and categorical variables (sex, family history, and comorbidities) were expressed as absolute values (n). Bivariate correlation analysis (Spearman coefficients) was used to evaluate relationships between independent variables, drainage patterns, and CEAP classifications. Associations between variables were also analyzed by logistic regression (chi-square or Fisher’s exact test).

Sample size was calculated considering a 10% error margin and a saphenous vein reflux prevalence of 32%. The ideal number of subjects was 84.

Statistical analysis was conducted using SPSS for Windows, version 22.0 (SPSS Statistics, Chicago, IL, United States) and the significance level was set at p < 0.05.

The protocol was approved by the institutional Ethics Committee (decision number 4145343) and signed consent was obtained from participants after detailed explanation of what the study involved, in accordance with Brazilian National Health Council resolution 466/2012 and the Helsinki Declaration.

## RESULTS

**Figure 1:**
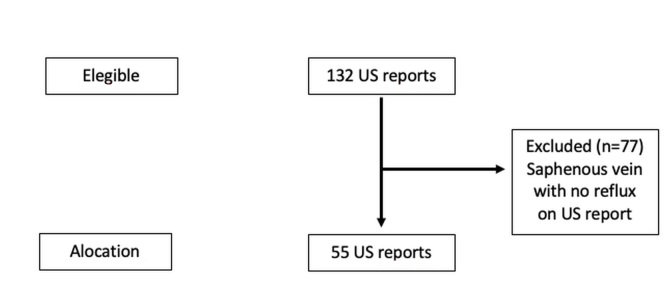
Flowchart of the patient sample identification and selection process.

A total of 55 reports were analyzed from ultrasound examinations of the lower limb venous system that met the inclusion criteria. A total of 132 patients had undergone ultrasound mapping prior to surgical procedures. Seventy-seven of these patients were excluded because of absence of saphenous vein reflux and the final sample comprised 55 subjects, as illustrated in Figure 1.

In the sample assessed, 73% were female, 27% were male, mean age was 56 years, and mean body mass index was 27. A sedentary lifestyle was identified in 53% of the sample.

With regard to CEAP classification, 20% were classified as C2, 53% as C3, and 27% as C4. With regard to time since onset, 35% had had symptoms for less than 5 years, 20% for 5 to 10 years, 22% for 10 to 20 years, and 13% of the patients had first had symptoms more than 20 years previously. Additionally, 65% of the sample reported a family history of varicose veins. Table 1 lists epidemiological data on the sample.

**Table 1:** Clinical and demographic data for the patients analyzed.

	**CEAP 1, 2, and 3**	**CEAP 4 and 5**	**Entire sample**
Sex			
Female	23	5	28
Male	5	3	8
Age (years)			
Mean (SD)	51.67 (16.9)	60.38 (7.4)	56.03 (6.8)
Associated diseases			
SAH	6	3	9
SAH + other disease	6	0	6
None	13	4	17
Others	2	1	3
Family history			
Yes	20	4	24
No	8	4	12
Time since onset			
< 5 years	12	1	13
5 to 10 years	5	2	7
10 to 20 years	4	4	8
> 20 years	7	1	8

SD = standard deviation; SAH = systemic arterial hypertension.

**Figure 2:**
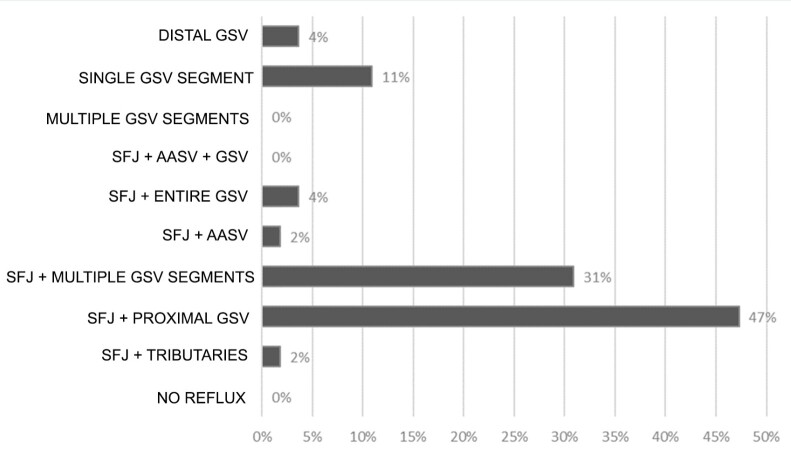
Great saphenous vein reflux patterns. GSV = great saphenous vein; SFJ = saphenofemoral junction; AASV = anterior accessory saphenous vein.

Characteristics of reflux in the great saphenous vein were assessed. In the whole sample, 47% of the patients had reflux at the saphenofemoral junction (SFJ) and the proximal great saphenous vein; 31% of the patients had reflux at the saphenofemoral junction and reflux in multiple segments of the saphenous vein; in 11% of the sample the great saphenous vein was incompetent in a single segment, without SFJ compromise, combined with reflux originating in tributary or perforating veins; 4% had distal great saphenous vein incompetence; another 4% had reflux originating at the saphenofemoral junction and a great saphenous vein that was incompetent along its entire length; 2% of the patients had reflux originating at the saphenofemoral junction and draining through tributary veins; and 2% had reflux at the saphenofemoral junction and the anterior accessory saphenous vein. Figure 2 illustrates the distribution of reflux patterns found in the ultrasound examination reports analyzed.

**Figure 3:**
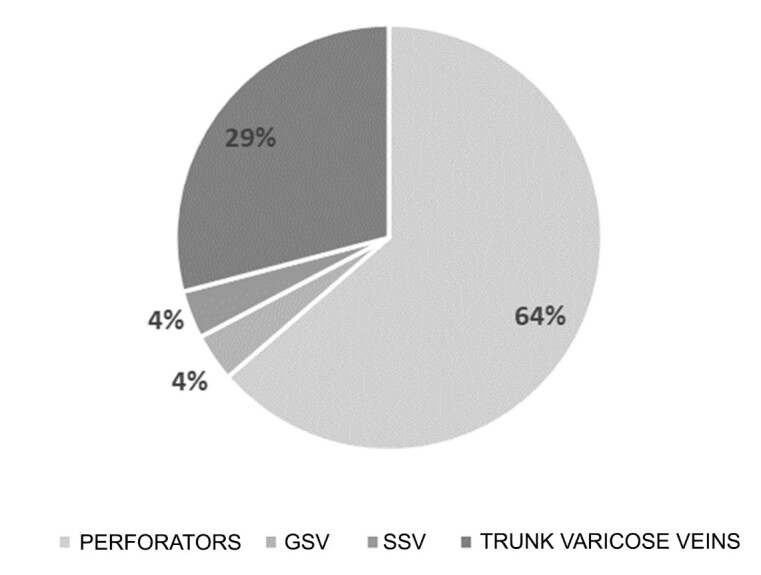
Distribution of types of drainage of flow from varicose veins originating in the great saphenous vein. GSV = great saphenous vein; SSV = small saphenous vein.

The distribution of drainage of reflux from varicose veins originating in the great saphenous vein was also analyzed. In this sample, 4% of the patients had varicose veins that drained to the great saphenous vein itself; in another 4%, drainage was to the small saphenous vein; in 29% drainage was to trunk varicose veins from which it was not possible to identify drainage to the deep vein system; and in 64% of the patients, varicose veins drained to perforating veins. Figure 3 illustrates the prevalence of types of drainage of reflux from the great saphenous vein to different sites.

The anatomic locations of the perforating veins were as follows: 3 (9%) were perforators of the medial aspect of the thigh (Hunter’s perforator); 13 (37%) perforated the medial aspect of the leg; 7 (20%) perforated the lateral aspect of the leg; 5 (14%) were pretibial perforators; and 7 (20%) were posterior calf perforators.

Considering drainage of calf perforating veins only, 13 (41%) drained to perforators of the medial aspect of the leg; 7 (22%) to perforators of the lateral aspect of the leg; 7 (22%) to perforators of the posterior calf; and 5 (16%) to pretibial perforators.

**Figure 4:**
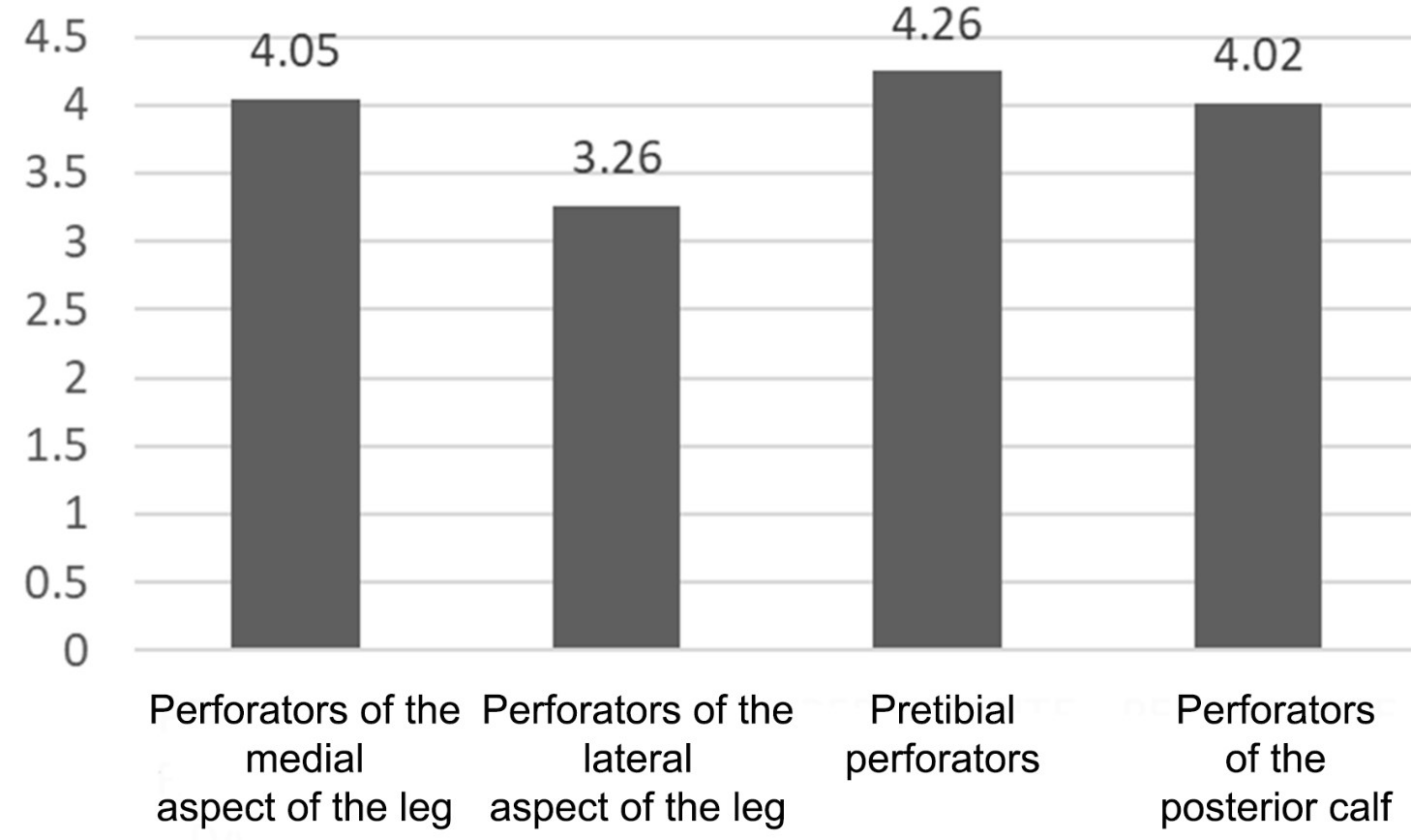
Mean diameters of perforating veins (mm).

The overall mean diameter of perforating veins was 3.92 mm and the mean diameters of each group of perforating veins are illustrated in Figure 4.

All of the patients reported symptoms and the feeling of tired legs was the most important symptom. Symptoms were graded with scores from 0 to 5, where zero means absence of the symptom and five indicates the worst intensity possible.

The correlation analysis demonstrated that there was a statistically significant positive correlation between the patients’ symptoms and CEAP classification. However, there were no significant correlations between drainage patterns, reflux patterns, or vein diameters and any of the symptoms (p > 0.05). Correlations between symptoms and the variables CEAP classification; types of GSV reflux; characteristics of varicose vein drainage; and diameters of perforating veins are shown in Table 2.

**Table 2:** Correlations between symptoms assessed, drainage patterns, and CEAP classification.

	**Pain**	**Heaviness**	**Tiredness**	**Burning**	**Swelling**
Drainage patterns	-0.063(0.63)	-0.011(0.94)	0.04(0.76)	-0.004(0.97)	0.202(0.11)
CEAP classification	0.39(0.002)*	0.38(0.002)*	0.212(0.095)	0.67(0.0004)^*^	0.45(0.0009)*
Reflux pattern	0.034(0.79)	0.073(0.57)	0.055(0.67)	0.079(0.54)	0.105(0.41)
Diameter	-0.018(0.92)	-0.001(0.99)	-0.024(0.90)	0.085(0.64)	0.130(0.48)

Spearman correlation (p).

* Significant correlation (p < 0.05).

Moreover, the multivariate linear regression showed that drainage patterns and reflux patterns could not be explained by symptoms in isolation or in association models (p > 0.05) (Table 3).

**Table 3:** Multivariate regression models for drainage pattern

**Model** ^a^	**Sum of squares**	**DF**	**F**	**p**
1	6.698	1	1.037	0.313^b^
2	18.600	2	1.460	0.240^c^
3	22.476	3	1.168	0.330^d^
4	22.575	4	0.865	0.490^e^
5	32.867	5	1.018	0.416^f^
6	44.193	6	1.156	0.343^g^

(a) Outcome variable: drainage patterns;

(b) Predictor: pain;

(c) Predictors: pain, heaviness;

(d) Predictors: pain, heaviness, tiredness;

(e) Predictors: pain, heaviness, tiredness, burning;

(f) Predictors: pain, heaviness, tiredness, burning, swelling;

(g) Predictors: pain, heaviness, tiredness, burning, swelling, CEAP classification;

DF: Degree of freedom; F: Variance ratio.

## DISCUSSION

Identification of specific great saphenous vein (GSV) reflux patterns using ultrasound with Doppler is one of the pillars of diagnosis and follow-up of chronic venous disease.^6^

Many studies of the subject have shown that reflux in superficial or deep veins in a specific general population can be of high prevalence and may be dependent on sex, age, and CEAP clinical stage.^3^,^4^,^7^,^8^

Saphenofemoral junction compromise is common and is caused by insufficiency of the terminal or pre-terminal valves, allowing reflux to the GSV and, consequently, to superficial collateral veins.^9^,^10^

Specifically regarding saphenous vein reflux, one of the most prevalent drainage patterns involves reflux at the SFJ and in the saphenous vein itself, as shown by published data available in the literature.^8^

In 86% of the sample, reflux was identified in the SFJ, most frequently in combination with reflux in the proximal segment of the GSV (47%) or with reflux in multiple GSV segments (31%). Saphenofemoral junction reflux was also observed in conjunction with reflux in the AASV (2%), with reflux in the entire GSV (4%), and with reflux in tributaries to the thigh (2%).

These findings regarding the saphenous vein reflux pattern differ from previous publications in the literature.^7^,^8^

One possible explanation for this data divergence could be the fact that our sample was based on patients who were referred for ultrasound mapping for varicose vein surgery. Furthermore, the presence of patients classified as C3 and C4 may increase the incidence of JSF and GSV reflux in this specific sample.

A study that analyzed GSV drainage patterns in patients classified as CEAP C2 also observed the prevalence of reflux involving the SFJ and the proximal GSV segment, finding a significantly higher prevalence of this pattern in males than females (p < 0.008).^7^

Engelhorn et al.^8^ conducted a prospective study analyzing 1,416 lower limbs, finding that 72% of the sample had reflux in the GSV. However, they found a higher prevalence of reflux compromising a single segment of the GSV, without any association with the SFJ.

They also analyzed the points of drainage of reflux from the GSV. The most frequently observed were in leg tributaries (33.97%), followed by thigh tributaries (28.68%), perforating leg veins (19.15%), knee tributaries (9.95%), perforators of the thigh (7.09%), and knee perforating veins (1.16%), equating to a total of 27.40% with drainage to perforating veins.^8^

In our study, there was a 64% prevalence of drainage of varicose vein reflux to perforating veins. Our interest in identifying the final destination of the flow from varicose veins may be the reason for the discrepancies between the study findings.

The characteristics of the samples may also be responsible for the discrepancies between study findings. Studies focused on CEAP classification C2^11^,^12^ differ from the present study, in which the majority of the sample were patients classified as CEAP C3. The natural progression of the disease can cause changes to drainage patterns and can also provoke anatomic changes such as increased diameters and extent of reflux.^6^

Many different questionnaires are available for quality-of-life assessment in patients with varicose veins of the lower limbs.^13^-^15^

In this study, patients were asked to rate their most frequent symptoms of the pathology. Studies have demonstrated a direct relationship between severity of chronic venous disease and deterioration of quality of life, essentially with respect to physical and functional aspects.^16^,^17^

In addition to increasing as CEAP grades increase, symptoms can also be linked with age group, since older patients have more comorbidities and greater physical limitations that can impact their quality of life.^18^

In this study, a positive and statistically significant correlation was observed between symptoms and CEAP classification. However, no similar positive relationship was observed with reflux pattern or perforating vein diameters, which did not have significant correlations with any of the symptoms (p > 0.05). The uniformity of the sample in terms of CEAP classification explains the lack of correlation between severity of symptoms and the characteristics of great saphenous vein reflux or drainage of varicose vein flow to the deep vein system.

This study contributes valuable information for understanding reflux drainage patterns from trunk varicose veins with origin in incompetent great saphenous veins. It is essential to differentiate between perforating veins providing drainage and perforating veins causing varicose veins, since the treatment for these two classes of perforators is different.

The diameters of perforating veins have been associated with their incompetence on Doppler ultrasound examination.^19^ Sandri et al.^19^ reported that 90% of perforating veins with diameters exceeding 3.5mm had reflux.

Presence of reflux in perforating veins may not in itself constitute a pathological vein state, especially not in veins located in the medial aspect of the leg.^20^

The mean diameter of perforating veins in our sample was 3.92mm, but despite having enlarged diameters, they were not associated with worse symptoms.

Since the sample analyzed presented with large varicose veins, these perforating vein diameters are understandable.

Perforating veins responsible for drainage of venous flow originating from varicose tributary veins of the saphenous vein dilate in order to perform the function of draining this blood to the deep vein system. When reflux from the great saphenous vein and its varicose tributaries is eliminated, spontaneous reduction of the diameters of the perforating veins is observed.^21^-^24^

The sample smaller than the ideal size calculated was a limitation of the present study.

In this study of a sample mostly composed of C3 patients, we found large perforating veins draining large varicose veins originating from refluxing saphenous veins, whose main role is to maintain the physiologic venous flow path towards the heart. There was no correlation with venous symptoms, which is compatible with the drainage feature of the perforating veins analyzed in this study.

## CONCLUSION

The most common pattern of drainage of the flow from varicose veins originating in an incompetent great saphenous vein to the deep system is through perforator veins. The prevalence of perforator veins in the sample was 64%, with no correlation with clinical symptoms.

Understanding the characteristics of drainage of venous reflux from varicose veins to the deep vein system through the perforating veins is crucial to appropriate clinical and surgical management of varicose veins of the lower limbs. Understanding the difference between perforating veins providing drainage and perforating veins associated with varicose veins is crucial to deciding the best intervention in each case.

Given that there are other populations with CVI, with different clinical presentations and different anatomical characteristics, we highlight that there is a need for further studies to ratify our findings in different populations from the one analyzed in our study.
